# A Sustainable Alternative for Green Structural Lightweight Concrete: Performance Evaluation

**DOI:** 10.3390/ma15238621

**Published:** 2022-12-02

**Authors:** Fahad K. Alqahtani

**Affiliations:** Department of Civil Engineering, College of Engineering, King Saud University, P.O. Box 800, Riyadh 11421, Saudi Arabia; bfahad@ksu.edu.sa

**Keywords:** sustainable structural lightweight concrete, ASTMC330/C330M-14, aggregates, physical, mechanical and chloride penetration resistance

## Abstract

The use of structural lightweight concrete in the construction industry is on the rise in the last few decades mainly because of the higher strength per unit density, as it reduces the total deal load of the structural elements as compared with normal strength concrete. In addition, the environmental concerns of the concrete industry have gained supreme importance in recent times, demanding vital and effectual steps. In this regard, the current study was carried out to formulate an alternative approach for producing a sustainable lightweight structural concrete. The study followed two stages: initially, the selection of optimized manmade plastic aggregates based on trial concrete mixes, and finally, to gauge the physical-, mechanical- and durability related properties of the concretes integrating optimized manmade aggregate series at different replacement fractions. As a result of the first phase: two aggregate series out of eight were selected based on the compressive strength and durability properties of their concretes. In the next stage, all the properties for the optimized aggregate concrete were analyzed in terms of compressive strength. It was noted that the physical, mechanical and chloride penetration resistances have generally displayed a decreasing trend, with an increase in the manmade plastic aggregate replacement fractions as compared with reference lightweight concrete. However, the two aggregates, i.e., 70% DS-30% LLDPE and 50% QF-50% PET at the replacement fractions of 25% and 100%, were found to be the best two contenders that fulfilled the criteria for structural lightweight concrete, i.e., ASTMC330/C330M-14, and were proposed for structural lightweight purposes with low and relatively high strength and chloride resistance-based durability requirements, respectively. In addition, the brittleness ratios and structural efficiency parameters for the concretes of the 70% DS-30% LLDPE and 50% QF-50% PET also supplemented the aforementioned findings. Overall, this study presents a sustainable approach for the effective utilization of plastic waste for producing structural lightweight concrete.

## 1. Introduction

Concrete construction has become one of the popular techniques all over the world, with its applications varying from a small single-family house to multi-story structures, long-span bridges and other mega-infrastructures such as dams, etc. The main reason for its popularity is the availability of raw materials for its components and well-established casting and curing procedures, along with its superior mechanical and durability properties against different environmental conditions compared with other available construction materials. However, with the advent of new and lightweight construction materials, one of the highlighted factors for normal concrete is the lower strength per unit density, which increases the size of structural elements.

Lightweight concrete has several intrinsic advantages such as lessening the total weight of a structure by a reduction in the structural elements’ sizes, which subsequently affects the usage of the two most expensive ingredients in reinforced concrete, i.e., steel reinforcement and cement. In addition, the lightweight concrete has remarkable sound and heat penetration resistance because of very low thermal conduction, mainly because of the presence of air voids as compared with conventional concrete. The density of normal concrete is mainly influenced by the density of the aggregates used in the concrete mix design. Therefore, one the suitable alternatives to producing lightweight concrete is to use lightweight aggregates to lower the density of the resulting concrete, as the aggregates occupy the maximum volume in the concrete. However, the incorporation of lightweight aggregates can negatively affect the physical, mechanical and durability properties of lightweight concrete, especially if the target is to use the resulting concrete for structural purposes.

In the past, a few researchers have tried to produce structurally lightweight concrete by using different materials as a replacement of normal coarse aggregates, i.e., expanded polystyrene (EPS) lightweight aggregate, organic lightweight aggregate, solid waste or recycled by-products and expanded clay aggregates [1,2,3,4]. A detailed experimental study was conducted to precisely estimate the compressive strength of structural lightweight concrete produced by using different types of expanded clays [5]. Zhang et al. [6] had made a structural lightweight concrete by using ordinary fine aggregates and expanded clays to evaluate its shrinkage performance. Scoria has also been used to produce structural lightweight concrete and compare the properties of the resulting concrete with ordinary Portland cement concrete [7].

In addition, a few efforts have been made in the past to use plastic waste for producing lightweight concrete [8,9,10,11,12,13,14,15,16], however, it was observed that the majority of these studies have focused on using the shredded pieces of recycled plastic as a replacement of natural fine or coarse aggregate. It was noticed that the concrete produced by using waste plastic as a replacement for normal aggregates had shown a negative impact on fresh and mechanical properties [8,9,11,14,17,18,19,20]. A lower workability (40% to 70%) of the concrete containing 20% of the polyethylene terephthalate (PET) as replacement to fine aggregates was observed [8,9].

The reduction in fresh density was observed for the replacement of coarse aggregate with expanded polystyrene [9]. The dry density decreased (65%) with 80% replacement of coarse aggregates with plastic particles, while a decrease ranging from 60 to 80% was observed in the mechanical strength when the coarse aggregates were replaced with plastic particles (from 15% to 80%) [8,9,11,14,17,18,19,20].

It was also reported that the recycling process, the type of plastic and properties of synthetic aggregates manufactured using plastic significantly affected the properties of the resulting lightweight concrete [21]. Additionally, maximum percentages of 10% and 20% were recommended for use as coarse aggregate in concrete for polypropylene and high-density polyethylene, respectively, however for non-structural purposes [22,23]. An M20-grade concrete was prepared by using polyethylene terephthalate plastic as replacement for fine aggregates [24]. In another study, M20-grade concrete was targeted by using a manufactured plastic-based coarse aggregate [25]. However, it was observed that the above-mentioned studies have formulated the concrete mainly for non-structural purposes.

In addition, the environmental concerns of the concrete industry have become of utmost importance in recent times and need urgent and efficient steps to lower the carbon footprints of the construction industry. Thus, in light of the above, the current experimental study was planned to formulate a sustainable green lightweight concrete for structural applications complying with ASTM C330/C330M-14 [26]. It was planned to achieve this target by using a manmade plastic-based aggregate, manufactured by using the technique registered by Alqahatni et al. (2021) [21]. The current study was conducted in two stages: In the first stage, the optimization of the manmade green aggregates was performed based on the performance of trail concrete mixes for the mechanical and durability parameters. The material’s availability was also considered while deciding the final aggregate series.

In the second stage, a detailed investigation was carried out for the concrete mixes prepared by the selected aggregates at various fractions, i.e., 25%, 50%, 75% and 100%, to examine the possibility of using the selected aggregate for manufacturing a concrete fulfilling ASTM C330/C330M-14 [26] criteria in the light of the analysis carried out for the concrete’s dry density, compressive strength, flexural strength, splitting tensile strength, modulus of elasticity, abrasion resistance, thermal conductivity and durability-related property (resistance to chloride ion). All the studied properties were analyzed in comparison with compressive strength as a common baseline along with the comparison with the already existing literature. The final outcome of this study provides an alternative sustainable approach to prepare a sustainable lightweight structural concrete. In addition, it will help to lower the carbon footprints of concrete along with the effective utilization of plastic waste.

## 2. Trail Mix, Optimization and Selection for Detailed Investigation

In the first stage of the research study, the trail mixes of concretes were prepared by keeping the majority of the parameters (such as water-to-cement ratio) constant, except the type of coarse aggregate. A total of ten concrete mixes were prepared using eight different types of manmade plastic aggregates and two control mixes having the lightweight aggregates (readily available in the market) and normal weight aggregates, as shown in Table 1. The parameters which differed in the manmade plastic aggregates were the type and percentage of plastic and filler material used. Two types of plastic (polyethylene terephthalate (PET) and linear low-density polyethylene (LLDPE)) and two types of fillers material (dune sand (DS) and quarry fines (QF)) at different percentages were used to manufacture the manmade plastic aggregates, as shown in Table 1. The major physical properties of DS and QF are shown in Table 2.

The optimized manmade plastic aggregate series was selected based on the performance of concrete mixes with regard to compressive strength, durability indicator, i.e., chloride ion penetration, and availability of the raw material. As seen from the results in Table 1, it was observed that compared with lightweight concrete, LLDPE-based aggregates show a decrease in the compressive strength for all types and percentages of filler materials, however, the percentage decrease is not significantly larger. Additionally, C1 and C3 show better durability performance, as indicated by the lower values of charge being passed through concrete specimens. Both C1 and C3 are included in the aggregate types manufactured using DS, and DS also has an advantage of being available in huge quantities free of charge (except the transportation), in addition to the environmental benefits which will further help to make the concrete more sustainable. Therefore, in light of the above, the manmade plastic aggregates used in C3 were selected from the LLDPE group, whereas, for the PET plastic type, C6 was selected, as it showed a significant rise in the compressive strength and also lower values for the charge passed through concrete as compared with the reference lightweight concrete. Thus, as a result, two aggregate series, i.e., the first made by 70% DS and 30% LLDPE (MM1) and the second made by 50% QF and 50% PET (MM2), were selected as the optimized manmade plastic aggregate series for further investigation.

## 3. Materials and Methodology

**Figure 1:**
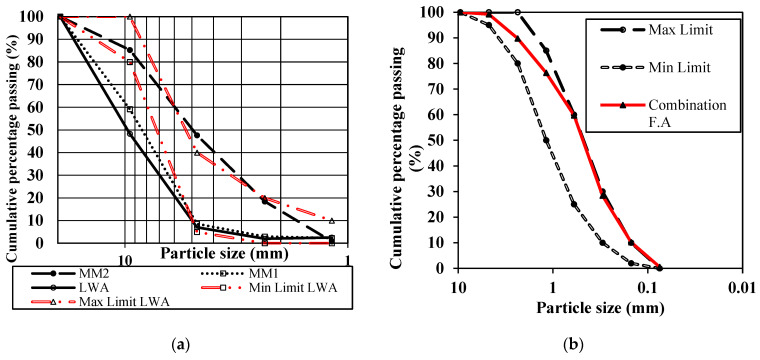
Particle size distribution for the (**a**) coarse aggregates and (**b**) fine aggregates.

For the second stage of the current study, the two optimized manmade plastic aggregates (MM1 and MM2) were used as coarse aggregate at different replacement ratios of 25%, 50%, 75% and 100%. In addition, for comparison purposes, a commercial lightweight (Lytag aggregates) and a normal-weight coarse aggregate were also used to prepare reference concrete mixes (LCC and NCC). Figure 1a shows the particle size distribution curves of the manmade plastic aggregates along with ASTM criteria limits for lightweight aggregates ASTM C136/C136M-14 [27]. The results of particle size distribution show that MM1 diverged a little with respect to the lower ASTM boundary, while MM2 was observed to nearly lie within the ASTM-defined boundaries. All coarse aggregate series were limited to the maximum size of 10 mm. From the economic and environmental point of view, it was decided in this study to utiliz dune sand mixed with crushed sand to fulfil the ASTM-defined criteria for fine aggregates, as shown in Figure 1b. The major physical properties of dune sand were already mentioned in the previous section in Table 2.

Ordinary Portland cement (Type I) having a specific gravity of 3.15 was utilized in the current study for the casting of concrete mixes. The dry unit weight for MM1A and MM2A was 750 kg/m^3^ and 1132 kg/m^3,^ respectively. The water absorption was found to be 2.75% and 0.95% for MM1A and MM2A, respectively. For the detailed investigation, ten concrete mixes were prepared with a constant water-to-cement ratio of 0.5, as shown in Table 3. MMXCY was the designation used in the table for eight of the concrete series. “X” represents the type of aggregate, i.e., MM1 (70% DS and 30% LLDPE) and MM2 (50% QF and 50% PET), while “Y” represents the weight replacement ratio, i.e., A, B, C and D for 25%, 50%, 75% and 100%, respectively. All the preparation, casting and curing procedures and methods were followed in accordance with ASTM C192/C192M-16 [28]. The total water quantity mentioned in the above table was assessed by considering the absorption values of the manmade plastic aggregate series.

**Figure 2:**
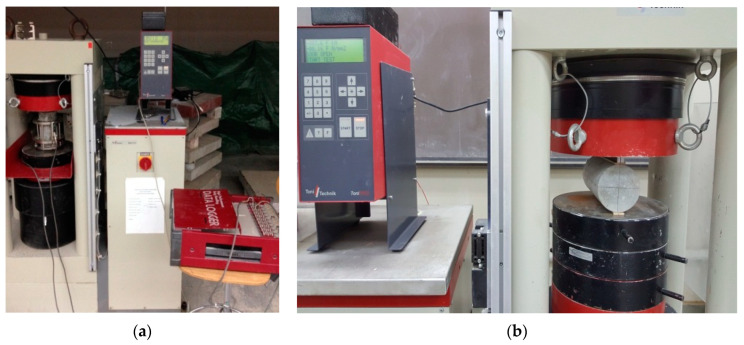
Setup for the compressive strength test (**a**) and splitting tensile strength test (**b**).

The dry density, compressive strength, flexural strength, splitting tensile strength, modulus of elasticity, abrasion resistance, thermal conductivity and resistance to chloride ion tests were conducted on all the concrete types. The test setup for the mechanical testing is shown in Figure 2 below. The entire range of tests conducted and their related standards are shown in Table 4. For the majority of the parameters, an average value of three tests was presented.

## 4. Results and Discussions

The following section presents the results of major physical-, mechanical- and durability-related properties for the concrete mixes prepared by using the two optimized manmade plastic aggregates at different replacement ratios. It should be noted here that the results for all the properties are expressed in terms of the compressive strength, as it is one of the most important parameters for concrete in general.

### 4.1. Physical Properties

#### 4.1.1. Dry Density

**Figure 3:**
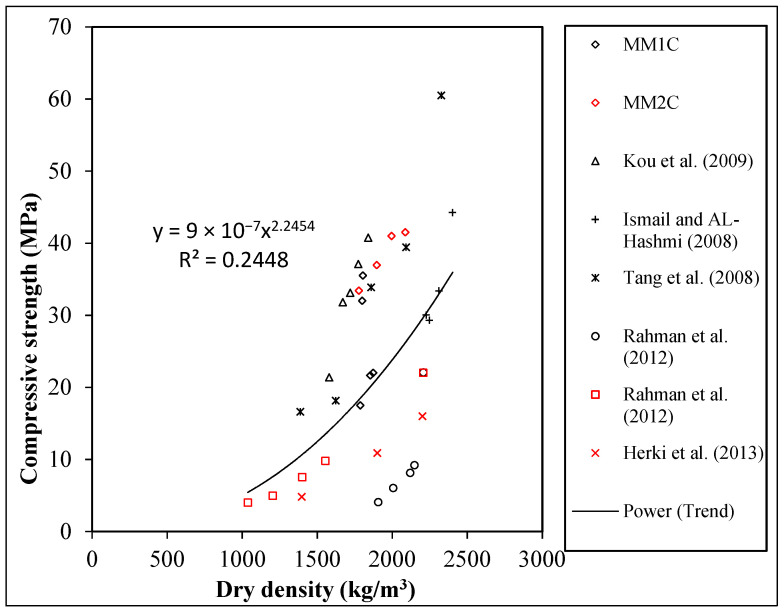
Relation between the cube compressive strength and dry density [9,11,15,37,38].

**Figure 4:**
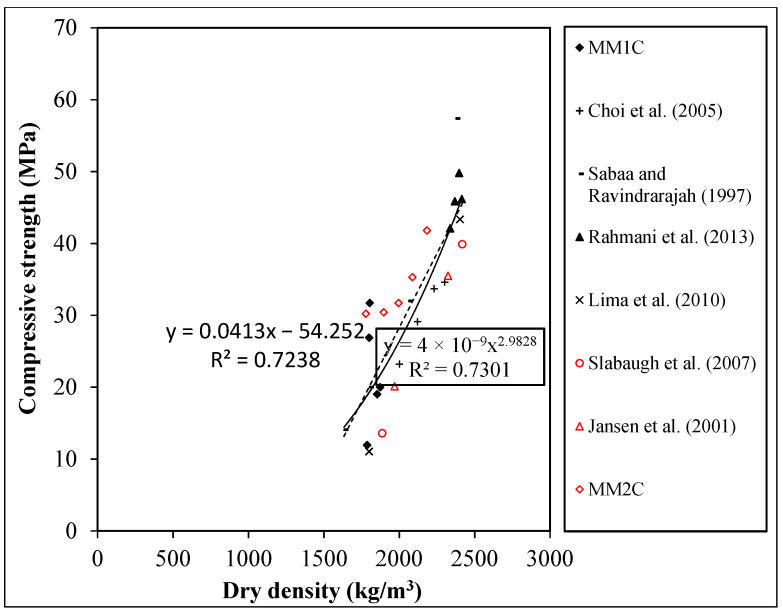
Relation between the cylindrical compressive strength and dry density [12,39,40,41,42,43].

The results for the dry density of MM1C and MM2C in relation to cube and cylinder compressive strength are presented in Figure 3 and Figure 4, respectively. It was observed that for MM1C, the increase in the percentage replacement of MM1 did not significantly affect the density, i.e., the maximum variation was 4% with respect to the control structural lightweight concrete, while a maximum decrease of about 19% was found for MMC2 series for 100% replacement with regard to NCC. It was observed that as the percentage of manmade plastic aggregates increased in the concrete matrix, both the density and compressive strength was found to decrease in general, as shown in Figure 3 and Figure 4. In addition, the data from the old studies for the density and the respective compressive for concretes where synthetic plastic aggregates were used were also plotted in Figure 3 and Figure 4 compared with the results of the current study. It was observed that density–cube compressive strength showed relatively larger variation as compared with the other studies [9,11,15,37,38]. In addition, a regression analysis was conducted, and an R^2^ value of 0.3078 was obtained for the best-fit equation, which also represented a greater divergence in the current study in comparison with other studies.

The cylinder compressive strength and density relation of the current study was also compared with the existing studies [12,39,40,41,42,43] as shown in Figure 4. It was observed that for the density–cylinder compressive strength, the results of the current study do not diverge significantly with respect to existing studies, as indicated by the R^2^ values of 0.663 (solid Line) and 0.724 (dotted line) for the power- and linear-based regression analyses. Overall, based on the results of density and cylinder compressive strength, the MM1A and MM2D qualified to be used as a structural lightweight concrete, as per the ASTMC330/C330M-14 [26] criteria.

#### 4.1.2. Abrasion Resistance

**Figure 5:**
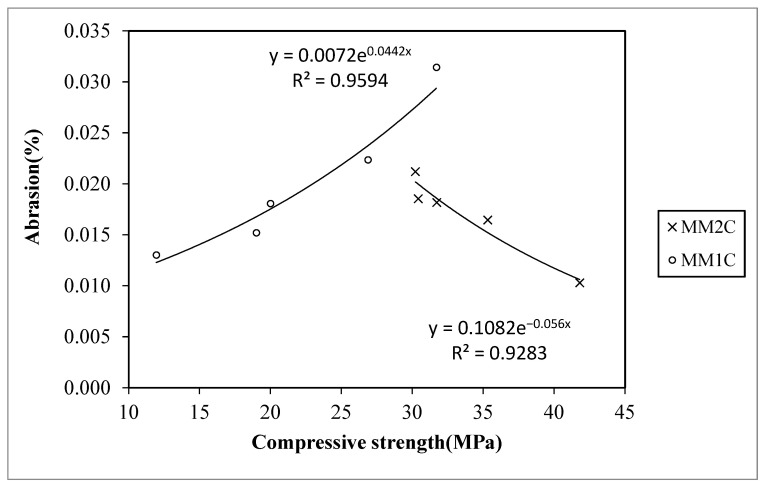
Relation between the cylinder compressive strength and abrasion resistance.

The trend of the abrasion resistance with respect to compressive strength for all concrete series is shown in Figure 5. It was observed that MMC1 and MMC2 show quite different behavior with an increase in the manmade plastic aggregate replacement ratio. It was observed that as the addition of aggregate replacement caused the compressive strength to decrease along with the abrasion, while for MM2C as the aggregate replacement ratio was raised, the compressive strength reduced with increased percentage of abrasion. The main difference between the behaviors is because of the plastic type and filler material being used for manmade plastic aggregates in each concrete type, which contributed to the difference in the texture of aggregate particles. The regression equation to predict the abrasion percentage in terms of compression strength is also shown in Figure 5. Table 5 shows the percentage difference between the predicted and experimental results by using the equations proposed in Figure 5. It was observed that the maximum difference was found to be less than 10%.

Overall, it was observed that the two series selected based on the density and compressive strength requirements, i.e., MM1A and MM2D, show almost the same values based on the results of abrasion and cylinder compressive strength, even with the different trend followed by their respective concrete series.

#### 4.1.3. Drying Shrinkage

**Figure 6:**
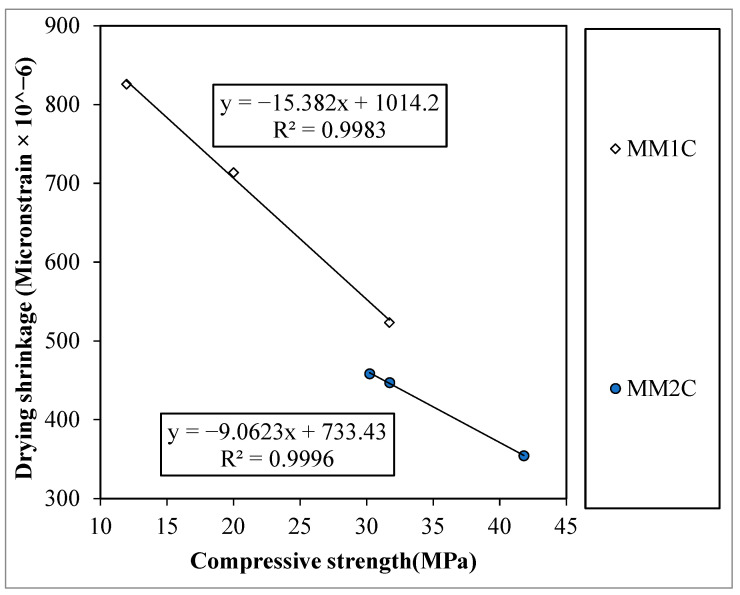
Relation between the cylinder compressive strength and drying shrinkage.

In this section, the author presents the relationship between the compressive strength and drying shrinkage of the concretes at various replacement percentages of manmade plastic aggregates. In addition, different plastic types were used for two plastic aggregates; therefore, to quantify the effect, the author performed the regression analysis and tried to formulate and equation based on the current experiment results. The trend of the drying shrinkage with respect to compressive strength for all concrete series is shown in Figure 6. It was observed that MMC1 and MMC2 show almost similar behavior with an increase in the manmade plastic aggregate replacement ratio.

It was observed that the addition of a plastic-based aggregate for MM1C and MM2C reduced the compressive strength, with a rise in the drying shrinkage. However, the increment in MM1C was found to be higher as compared with MM2C. This behavior of a rise in drying shrinkage in concrete containing manmade plastic aggregate was expected, as the primary factor influencing it is the amount of water loss, which in the case of the aforementioned concrete will just be from the matrix alone, as the plastic aggregates will not contribute in this phenomenon. The equation to predict the drying shrinkage for manmade plastic aggregate concrete in terms of compressive strength is also proposed in Figure 6. Table 6 shows the percentage difference between the predicted and experimental results by using the equations proposed in Figure 6. It was observed that the maximum difference was found to be less than 2%.

### 4.2. Mechanical Properties

#### 4.2.1. Splitting Tensile Strength

**Figure 7:**
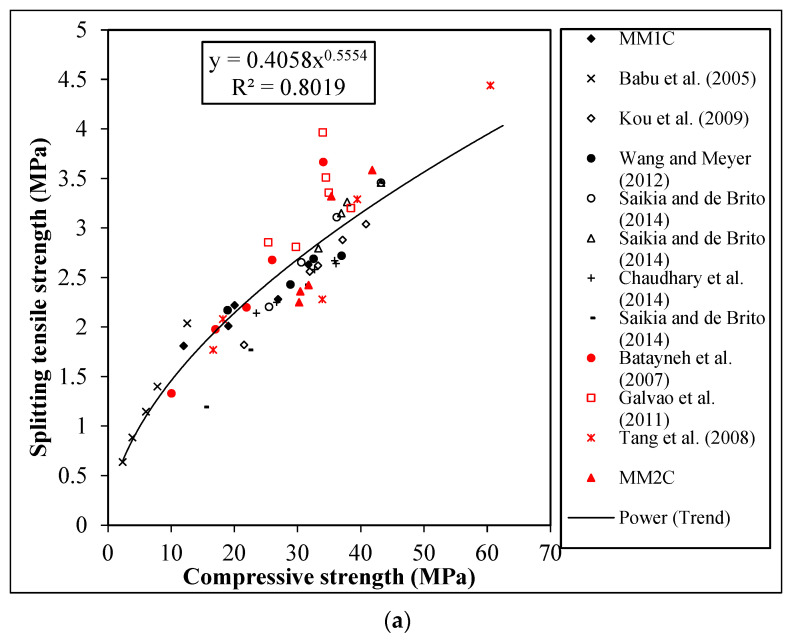

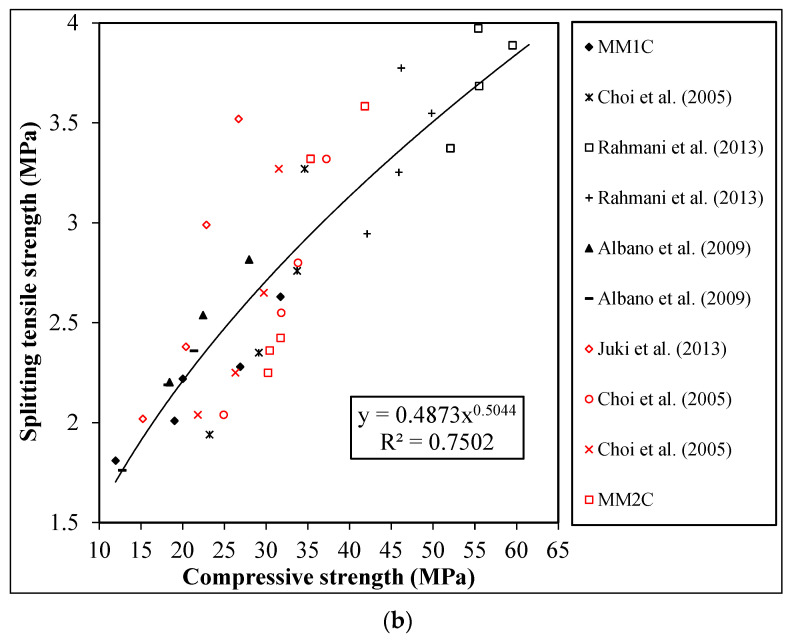
Relation between the tensile strength and (**a**) cube compressive strength [11,14,15,16,44,45,46,47,48]; (**b**) cylinder compressive strength [8,10,12,39].

The relation between the tensile strength and compression of normal concrete is well documented in the codes, however, the effect of incorporating the manmade plastic aggregates in tensile strength in relation to compressive strength is quantified in this section. The author measured the compressive strength of the concretes by using the cylindrical and cube specimens. In the past, both cube and cylindrical compressive strength has been used by researchers; therefore, the author presented the relation between tensile and the compressive strength measured by two differently shaped specimens to eliminate the shape effect of specimens, keeping the main variable parameter as the plastic replacement. The trend of the tensile strength with respect to cube and cylinder compressive strength for all concrete series is shown in Figure 7a,b, respectively. It was observed that irrespective of the aggregate type, the decrease in compressive strength was accompanied by a decrease in tensile strength, with an increase in the percentage replacement of aggregate series. However, the percentage decrease between the compressive and tensile strength of concrete for each replacement level of aggregate was found to be different. The maximum decreases for MM1C and MM2C were found to be 31% and 37%, respectively, with reference to the control mixes.

From Figure 7, it was observed that the current experiment study results are aligned with the existing literature. In addition, it was also observed that the cube compressive strength showed better correlation with the tensile strength of concrete containing manmade plastic aggregates in comparison with the cylinder compressive strength as represented by the R^2^ values of 0.8521 (Cube-Tensile) and 0.7456 (Cylinder-Tensile). Furthermore, the equations proposed in Figure 7a,b were used to predict the tensile strength of concrete containing manmade plastic aggregates, and the percentage difference between predicted and experimental results is shown in Table 7.

Overall, based on the results of tensile strength, density and cylinder compressive strength, MM1A and MM2D qualified to be used as a structural lightweight concrete as per the ASTMC330/C330M-14 [26] criteria.

#### 4.2.2. Bending Strength

**Figure 8:**
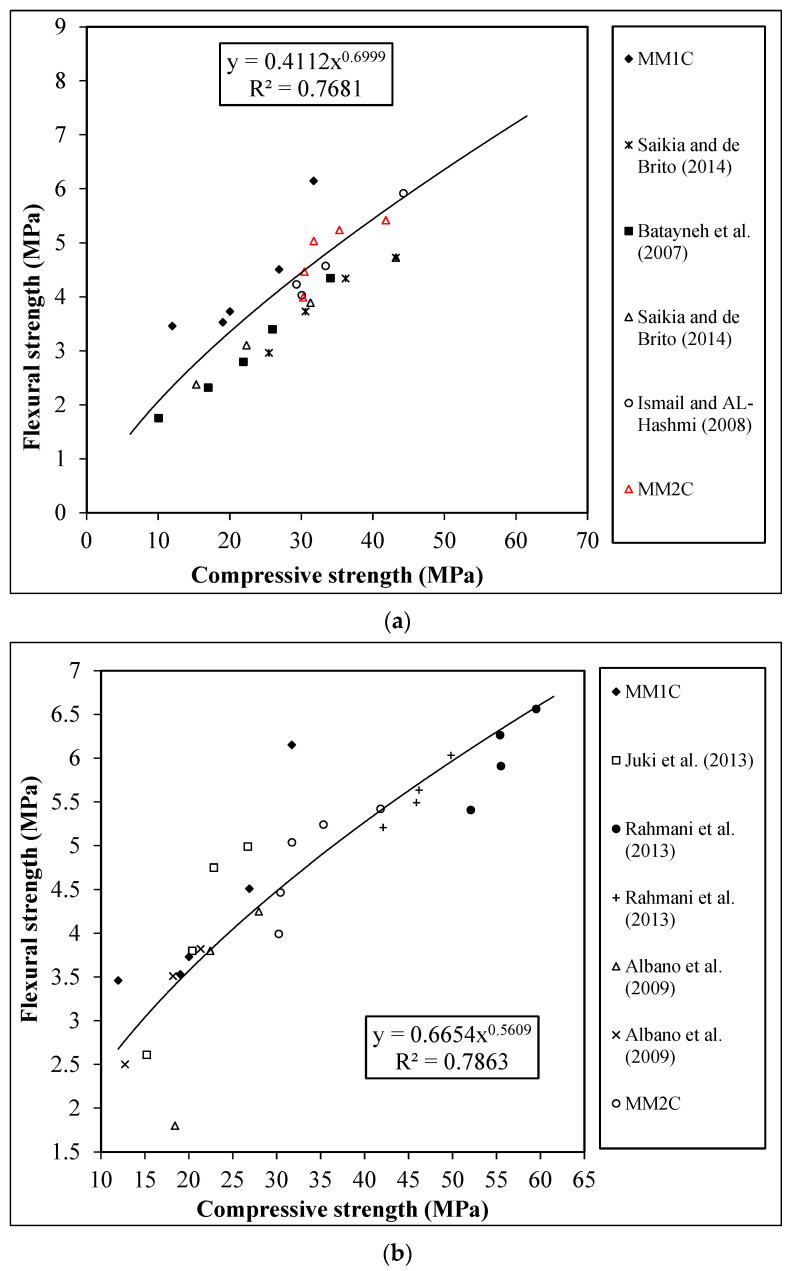
Relation between the bending strength and (**a**) cube compressive strength [9,45,47]; (**b**) cylinder compressive strength [8,10,12].

The trend of the bending strength with respect to cube and cylinder compressive strength for all concrete series is shown in Figure 8a,b, respectively.

It was observed that irrespective of the aggregate type, the decrease in compressive strength was accompanied by a decrease in the bending strength at increasing percentage replacement of aggregate series. However, the decrease ratio between the compressive and bending strength of concrete for each replacement level of aggregate was found to be different. The maximum decrease for MM1C and MM2C was found to be 44% and 26%, respectively, with reference to control mixes. From Figure 8, it is observed that the current experiment study results are aligned with the existing literature. In addition, like tensile strength, it was also observed that cube compressive strength showed better correlation with the bending strength of concrete containing manmade plastic aggregates in comparison with cylinder compressive strength, as represented by the R^2^ values of 0.8123 (cube-bending) and 0.7126 (cylinder-bending). Furthermore, the equations proposed in Figure 8a,b were used to predict the bending strength of concrete containing manmade plastic aggregates, and the percentage difference between predicted and experimental results is shown in Table 8.

### 4.3. Durability Indicator

**Figure 9:**
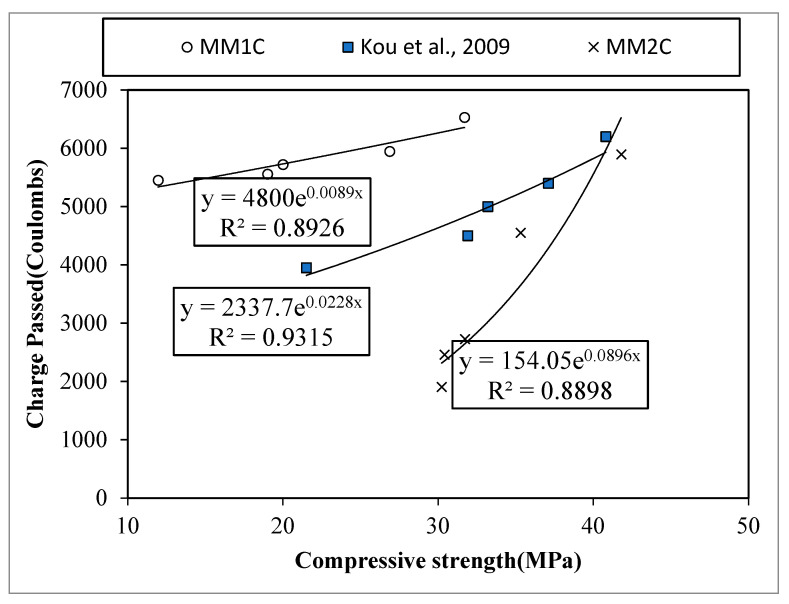
Relation between chloride penetration resistance and cylinder compressive strength [11].

In the current study, chloride penetration resistance was used as an indicator to access the durability and microstructure of the concrete containing different percentages of plastic aggregates. The trend of the chloride penetration resistance with respect to cylinder compressive strength for all concrete series is shown in Figure 9.

It was observed that irrespective of the concrete type, the higher the concrete strength, the higher the amount of charge passed; however, the slopes of both the concretes (MM1C and MM2C) were different. It was observed that at lower strength values, MM1C showed higher values of charge passed as compared with MM2C, while the difference seemed to narrow down with increasing values of compressive strength. Decreases of about 18% and 73% in the amount of charge passed were observed for MM1CD and MM2CD, respectively, as compared with LCC. All the concrete series except for MM2CD showed values of charge being passed through concrete greater than 2000 coulombs, which refer to the classification of the concrete permeability ranging from medium to high, as per ASTM C1202 [35]. Therefore, in light of the results of concrete durability, it was noticed that MM1CA has the potential to be used as structural lightweight concrete for projects having relatively low contact with chloride ions. On the other hand, MM2CD proved itself to be a sound candidate for all types of structural lightweight concrete applications irrespective of chloride levels; however, it should be noted here that a detailed experimental investigation is required to determine the durability properties (sulfate resistance, alkali–silica reaction, carbonation, etc.) of plastic-based aggregate concrete before its application in the field.

### 4.4. Discussion

**Figure 10:**
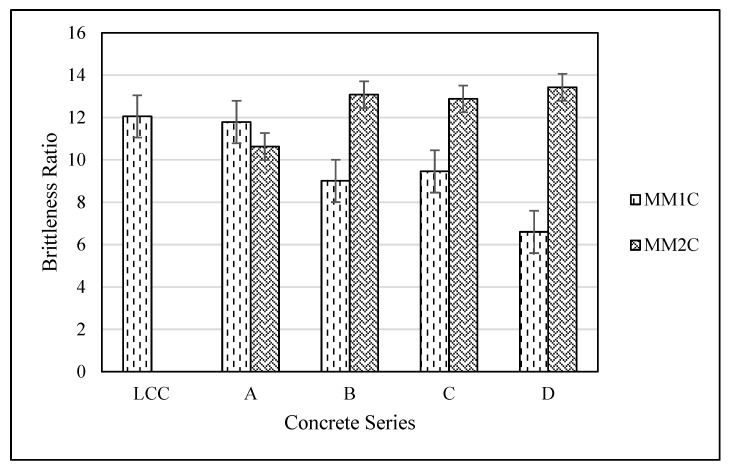
Brittleness ratio for all the concrete series.

In the preceding sections, the physical-, mechanical- and durability-related properties of concrete containing two different types of manmade plastic aggregates at various fractions are presented. The primary aim was to determine a suitable candidate for sustainable structural lightweight concrete. As plastic was used in the manmade aggregate, it was therefore expected that the concrete brittleness behavior would be improved because of the ductile nature of plastic. In this regard, the brittleness ratio, as defined by Hucka and Das (1974) [49], i.e., the compressive to tensile strength of a given concrete, was calculated for all the concrete series for the current study, as shown in Figure 10.

It was observed that MM1C concrete series show an inverse relationship between the percentage of MM1A and brittleness ratio, i.e., as the amount of MM1 aggregates surged, the concrete became less brittle as compared with the reference lightweight concrete. On the other hand, MM2C showed a marginal increment in the brittleness ratio with the addition of MM2A aggregate type as compared with LCC. This difference is primarily because of the difference in the type of plastic and filler material being used for the manufacturing of both types of aggregates. It was noticed that MM1C can be used in applications with low strength and high ductility, while MM2C can be targeted for relatively higher strength with moderate ductility requirements.

**Figure 11:**
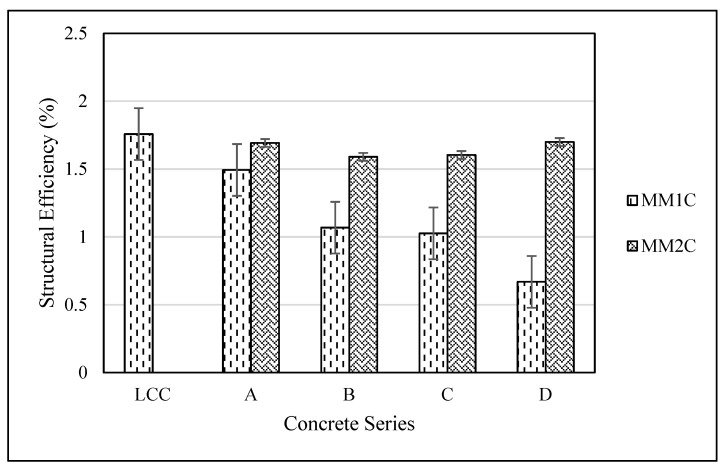
Structural efficiency for all the concrete series.

In addition, another important factor which is normally discussed for structural lightweight concrete is the structural efficiency. The structural efficiency is defined as the ratio between the cylindrical compressive strength and density of the given concrete as per ACI 213R-14 (2014) [50]. The structural efficiency values for the concretes cast in the current study are shown in Figure 11.

It was observed that for MM1C series, structural efficiency dropped with the increasing percentage of manmade plastic aggregates. The decrease was mainly because of the decrease in the compressive strength values. On the other hand, MM2C showed marginal decreases for 50% and 75% replacements of manmade plastic aggregates, and it was found to increase at the 100% replacement level, almost equivalent to that of the reference lightweight concrete. Therefore, from the structural efficiency aspect, MM2CD also showed its capacity to replace the commercially available lightweight aggregate. From the above discussion, it can also be deduced that replacing the commercially available lightweight aggregate, or natural lightweight aggregates, with the produced aggregates can effectively lower the carbon footprints of concrete and, in parallel, will help the utilization of plastic waste, as the aggregates occupy 70% of the concrete volume, and therefore a larger quantity can be replaced.

## 5. Conclusions

This study aimed to formulate a sustainable green lightweight concrete for structural applications complying with ASTM C330/C330M-14 [26]. It was planned to achieve this target by using a manmade plastic-based aggregate, manufactured by using the technique registered by Alqahatni et al. (2021) [21].

Therefore, this study was carried in two stages, i.e., the selection of optimized manmade plastic aggregates based on trial concrete mixes and the evaluation of the possibility of using the optimized aggregate series for structural lightweight construction applications. In this regard, the physical-, mechanical- and durability-related properties of the concretes integrating optimized manmade aggregate series were assessed, keeping compressive strength as the primary datum. The following conclusions are drawn:

1. Out of eight manmade plastic aggregates having different plastic and filler types, two, i.e., the first made by 70% DS and 30% LLDPE (MM1) and the second made by 50% QF and 50% PET (MM2), were selected as the optimized aggregate series based on the performance of concrete mixes with regard to compressive strength, durability indicator, i.e., chloride ion penetration, and availability of the raw material.
2. The results of the physical properties analyzed in terms of compressive strength give rise to the best two contenders, i.e., MM1 and MM2 at 25% and 100% replacement fractions, respectively. On the other hand, the mechanical properties generally displayed a decreasing trend with increase in the manmade plastic aggregate replacement fractions; however, MM1 and MM2 at 25% and 100% replacement fractions, respectively, fulfilled the criteria for structural lightweight concrete, i.e., ASTMC330/C330M-14 [26].
3. From the results of chloride ion penetration resistance, it was found that the aggregates made by 70% DS and 30% LLDPE were suitable for the projects having relatively low contact with chloride ions, while the aggregates made by 50% QF and 50% PET can be used for structural lightweight concrete applications irrespective of chloride levels. The brittleness ratio and structural efficiency also supported the use of MM1CA and MM2CD for structural lightweight concrete applications.
4. Finally, two aggregates, i.e., 70% DS-30% LLDPE and 50% QF-50% PET at the replacement fractions of 25% and 100%, were proposed for structural lightweight purposes with low and relatively high strength and chloride-based durability requirements, respectively. As a whole, the current study provides an alternative approach to target the sustainable ways for producing lightweight structural concrete by the utilization of plastic waste.

## Figures and Tables

**Table 1:** Trail mixes and their significant properties.

Concrete Type	Green Manmade Plastic Aggregate				Mechanical Aspect	Durability Aspect
	Plastic Type	Plastic Percentage	Filler Type	Filler Percentage	Compressive Strength (MPa)	Chloride Ion Resistance (Columns)
Normal Weight	-	-	-	-	43.70	5673
Lightweight	-	-	-	-	30.09	5811
C1	LLDPE	50	DS	50	15.48	5532
C2	LLDPE	50	QF	50	17.58	5859
C3	LLDPE	30	DS	70	15.24	5227
C4	LLDPE	30	QF	70	18.02	5875
C5	PET	50	DS	50	35.92	3996
C6	PET	50	QF	50	39.09	3612
C7	PET	30	DS	70	25.67	3647
C8	PET	30	QF	70	31.74	3417

**Table 2:** Major Physical properties of fillers used in this study.

Properties	Filler Type	
	DS	QFF
Color	Reddish	Creamy Whitish
Specific gravity	2.62	2.71
Median size (µm)	215	19.27
Absorption (%)	0.38	1.52
Shape	Irregular	Angular
Density (kg/m^3^)	1649	1531

**Table 3:** Mix proportions for concrete mixes used in the current study.

Concrete Type	W/C	Total Water	Free Water	Cement	Fine Aggregate	Coarse Aggregate		
						N	L	MMP
		kg/m^3^						
LCC	0.50	296.2	225	450	922	-	352	-
MM1CA		282.4			918	-	264	95
MM1CB		269.6			913	-	176	189
MM1CC		255.6			909	-	88	284
MM1CD		241.1			906	-	-	378
NCC		240.3			880	688	-	-
MM2CA		239			847	516	-	141
MM2CB		237.6			815	344	-	282
MM2CC		236.2			782	172	-	423
MM2CD		234.8			750	-	-	565

**Table 4:** Tested properties and their related standards.

Sr. No	Parameter	Procedure Adopted
1	Dry density	BS EN 12390-7:2009 [29]
2	Cylinder compressive strength	ASTM C39/C39M-16 [30]
3	Flexural strength	ASTM C580-02 [31]
4	Splitting tensile strength	ASTM C496/C496M-11 [32]
5	Modulus of elasticity	ASTM C469/C469M-14 [33]
6	Abrasion resistance	ASTM C944/C944M-12 [34]
7	Chloride permeability	ASTM C1202-12 [35]
8	Thermal conductivity	ASTM C177-13 [36]

**Table 5:** Difference between predicted and experimental results for abrasion.

Concrete Type	Predicted Values by Using the Figure 4 Eqs.		Percentage Difference between Predicted and Experimental Results
LCC	0.029	-	−7.89
MM1CA	0.023	-	4.75
MM1CB	0.017	-	−4.19
MM1CC	0.017	-	9.04
MM1CD	0.012	-	−6.61
NCC	-	0.011	2.73
MM2CA	-	0.015	−7.72
MM2CB	-	0.019	1.97
MM2CC	-	0.020	7.46
MM2CD	-	0.020	−4.99

**Table 6:** Difference between predicted and experimental results for drying shrinkage.

Concrete Type	Predicted Values by Using the Figure 5 Eqs. (Micronstrain × 10^−6^)		Percentage Difference between Predicted and Experimental Results
LCC	526.59	-	0.58
MM1CC	706.56	-	−1.02
MM1CD	830.39	-	0.53
NCC	-	354.63	0.04
MM2CC	-	445.97	−0.26
MM2CD	-	459.57	0.22

**Table 7:** Difference between predicted and experimental results for tensile strength.

Concrete Type	Predicted Values by Using the Equation in		Percentage Difference between Predicted and Experimental Results	
	Figure 6a	Figure 6b	Figure 6a	Figure 6b
LCC	2.63	2.79	5.92	5.00
MM1CA	2.28	2.56	12.42	10.51
MM1CB	2.22	2.21	−0.53	−3.69
MM1CC	2.01	2.15	7.07	3.41
MM1CD	1.81	1.70	−5.91	−11.25
NCC	3.58	3.20	−10.64	−10.15
MM2CA	3.32	2.94	−11.42	−11.70
MM2CB	2.42	2.79	14.92	13.93
MM2CC	2.36	2.73	15.55	14.30
MM2CD	2.25	2.72	20.85	19.51

**Table 8:** Difference between predicted and experimental results for tensile strength.

Concrete Type	Predicted Values by Using the Equation in		Percentage Difference between Predicted and Experimental Results	
	Figure 7a	Figure 7b	Figure 7a	Figure 7b
LCC	4.62	4.62	−24.81	−24.88
MM1CA	4.22	4.12	−6.54	−8.74
MM1CB	3.57	3.35	−4.26	−10.27
MM1CC	3.47	3.23	−1.68	−8.51
MM1CD	2.68	2.33	−22.68	−32.54
NCC	5.40	5.61	−0.37	3.45
MM2CA	4.91	4.98	−6.25	−4.92
MM2CB	4.63	4.62	−8.17	−8.24
MM2CC	4.52	4.49	1.15	0.48
MM2CD	4.50	4.47	12.72	11.88

## References

[1] Wang J., Zheng K., Cui N., Cheng X., Ren K., Hou P., Feng L., Zhou Z., Xie N. (2020). Green and Durable Lightweight Aggregate Concrete: The Role of Waste and Recycled Materials. Materials.

[2] Felicetti R., Gambarova P.G., Bamonte P. (2012). Thermal and mechanical properties of light-weight concrete exposed to high temperature. Fire Mater..

[3] Xu Y., Jiang L., Xu J., Li Y. (2012). Mechanical properties of expanded polystyrene lightweight aggregate concrete and brick. Constr. Build. Mater..

[4] Cheng C.M., Su D.G., He J., Jiao C.J. (2011). Compressive Strength of Organic Lightweight Aggregate Concrete. Adv. Mater. Res..

[5] Bogas J.A., Gomes M.G., Gomes A. (2013). Compressive strength evaluation of structural lightweight concrete by non-destructive ultrasonic pulse velocity method. Ultrasonics.

[6] Zhang M.-H., Dang L., Paramasivam P. (2005). Shrinkage of high-strength lightweight aggregate concrete exposed to dry environment. ACI Mater. J..

[7] Kılıç A., Atiş C.D., Yaşar E., Özcan F. (2003). High-strength lightweight concrete made with scoria aggregate containing mineral admixtures. Cem. Concr. Res..

[8] Albano C., Camacho N., Hernández M., Matheus A., Gutiérrez A. (2009). Influence of content and particle size of waste pet bottles on concrete behavior at different w/c ratios. Waste Manag..

[9] Ismail Z.Z., Al-Hashmi E.A. (2008). Use of waste plastic in concrete mixture as aggregate replacement. Waste Manag..

[10] Juki M.I., Awang M., Annas M.M.K., Boon K.H., Othman N., Kadir A.B.A., Roslan M.A., Khalid F.S. (2013). Relationship between Compressive, Splitting Tensile and Flexural Strength of Concrete Containing Granulated Waste Polyethylene Terephthalate (PET) Bottles as Fine Aggregate. Adv. Mater. Res..

[11] Kou S.C., Lee G., Poon C.S., Lai W.-L. (2009). Properties of lightweight aggregate concrete prepared with PVC granules derived from scraped PVC pipes. Waste Manag..

[12] Rahmani E., Dehestani M., Beygi M.H.A., Allahyari H., Nikbin I.M. (2013). On the mechanical properties of concrete containing waste PET particles. Constr. Build. Mater..

[13] Rai B., Rushad S.T., Kr B., Duggal S.K. (2012). Study of Waste Plastic Mix Concrete with Plasticizer. ISRN Civ. Eng..

[14] Saikia N., de Brito J. (2014). Mechanical properties and abrasion behaviour of concrete containing shredded PET bottle waste as a partial substitution of natural aggregate. Constr. Build. Mater..

[15] Tang W., Lo Y., Nadeem A. (2008). Mechanical and drying shrinkage properties of structural-graded polystyrene aggregate concrete. Cem. Concr. Compos..

[16] Wang R., Meyer C. (2012). Performance of cement mortar made with recycled high impact polystyrene. Cem. Concr. Compos..

[17] Hannawi K., Kamali-Bernard S., Prince W. (2010). Physical and mechanical properties of mortars containing PET and PC waste aggregates. Waste Manag..

[18] Marzouk O.Y., Dheilly R., Queneudec M. (2007). Valorization of post-consumer waste plastic in cementitious concrete composites. Waste Manag..

[19] Akçaözoğlu S., Atiş C.D., Akçaözoğlu K. (2010). An investigation on the use of shredded waste PET bottles as aggregate in lightweight concrete. Waste Manag..

[20] Frigione M. (2010). Recycling of PET bottles as fine aggregate in concrete. Waste Manag..

[21] Alqahtani F.K., Rashid K., Zafar I., Khan M.I., Ababtain A.A. (2021). Production of sustainable green mortar by ultrahigh utilization of fly ash: Technical, economic and environmental assessment. Constr. Build. Mater..

[22] Islam M.J., Shahjalal M. (2021). Effect of polypropylene plastic on concrete properties as a partial replacement of stone and brick aggregate. Case Stud. Constr. Mater..

[23] Abu-Saleem M., Zhuge Y., Hassanli R., Ellis M., Rahman M., Levett P. (2021). Evaluation of concrete performance with different types of recycled plastic waste for kerb application. Constr. Build. Mater..

[24] Bamigboye G.O., Tarverdi K., Umoren A., Bassey D.E., Okorie U., Adediran J. (2021). Evaluation of eco-friendly concrete having waste PET as fine aggregates. Clean. Mater..

[25] Castillo E.R., Almesfer N., Saggi O., Ingham J.M. (2020). Light-weight concrete with artificial aggregate manufactured from plastic waste. Constr. Build. Mater..

[26] (2014). Standard Specification for Lightweight Aggregates for Structural Concrete.

[27] (2014). Standard Test Method for Sieve Analysis of Fine and Coarse Aggregates.

[28] (2016). Standard Practice for Making and Curing Concrete Test Specimens in the Laboratory.

[29] (2009). Testing Hardened Concrete: Density of Hardened Concrete.

[30] (2016). Standard Test Method for Compressive Strength of Cylindrical Concrete Specimens.

[31] (2012). Standard Test Method for Flexural Strength and Modulus of Elasticity of Chemical-Resistant Mortars, Grouts, Monolithic Surfacings, and Polymer Concretes.

[32] (2011). Standard Test Method for Splitting Tensile Strength of Cylindrical Concrete Specimens.

[33] (2014). Standard Test Method for Static Modulus of Elasticity and Poisson’s Ratio of Concrete in Compression.

[34] (2012). Standard Test Method for Abrasion Resistance of Concrete or Mortar Surfaces by the Rotating-Cutter Method.

[35] (2012). Standard Test Method for Electrical Indication of Concrete’s Ability to Resist Chloride Ion Penetration.

[36] (2013). Standard Test Method for Steady-State Heat Flux Measurements and Thermal Transmission Properties by Means of the Guarded-Hot-Plate Apparatus.

[37] Rahman M.M., Islam M.A., Ahmed M. Recycling of waste polymeric materials as a partial replacement for aggregate in concrete. Proceedings of the International Conference on Chemical, Environmental and Biological Sciences (ICCEBS’12) 2012.

[38] Herki A., Khatib J., Negim E. (2013). Lightweight concrete made from waste polystyrene and fly ash. World Appl. Sci. J..

[39] Choi Y.-W., Moon D.-J., Chung J.-S., Cho S.-K. (2005). Effects of waste PET bottles aggregate on the properties of concrete. Cem. Concr. Res..

[40] Ravindrarajah R.S. Bearing strength of concrete containing polystyrene aggregate. Proceedings of the RILEM 8th International Conference Durability of Building Materials and Components.

[41] Lima P., Leite M., Santiago E.Q.R. (2010). Recycled lightweight concrete made from footwear industry waste and CDW. Waste Manag..

[42] Slabaugh S., Swan C., Malloy R. Development and properties of Foamed synthetic Lightweight Aggregates. Proceedings of the World of Coal Ash Conference.

[43] Jansen D.C., Kiggins M.L., Swan C.W., Malloy R.A., Kashi M.G., Chan R.A., Javdekar C., Siegal C., Weingram J. (2001). Lightweight Fly Ash-Plastic Aggregates in Concrete. Transp. Res. Rec. J. Transp. Res. Board.

[44] Babu D.S., Babu K.G., Wee T. (2005). Properties of lightweight expanded polystyrene aggregate concretes containing fly ash. Cem. Concr. Res..

[45] Saikia N., Brito J d. (2013). Waste polyethylene terephthalate as an aggregate in concrete. Mater. Res..

[46] Chaudhary M., Srivastava V., Agarwal V. (2014). Effect of waste low density polyethylene on mechanical properties of concrete. J. Acad. Ind. Res..

[47] Batayneh M., Marie I., Asi I. (2007). Use of selected waste materials in concrete mixes. Waste Manag..

[48] Galvão J.C.A., Portella K.F., Joukoski A., Mendes R., Ferreira E.S. (2011). Use of waste polymers in concrete for repair of dam hydraulic surfaces. Constr. Build. Mater..

[49] Hucka V., Das B. (1974). Brittleness determination of rocks by different methods. Int. J. Rock Mech. Min. Sci. Geomech. Abstr..

[50] (2003). Guide for Structural Lightweight-Aggregate Concrete-ACI Committee 213.

